# From Transcriptome to Noncoding RNAs: Implications in ALS Mechanism

**DOI:** 10.1155/2012/278725

**Published:** 2012-06-17

**Authors:** Stella Gagliardi, Pamela Milani, Valentina Sardone, Orietta Pansarasa, Cristina Cereda

**Affiliations:** ^1^Laboratory of Experimental Neurobiology, IRCCS National Neurological Institute “C. Mondino,” 27100 Pavia, Italy; ^2^Department of Neurological Sciences, University of Pavia, 27100 Pavia, Italy

## Abstract

In the last years, numerous studies have focused on understanding the metabolism of RNA and its implication in disease processes but abnormal RNA metabolism is still unknown. RNA plays a central role in translating genetic information into proteins and in many other catalytic and regulatory tasks. Recent advances in the study of RNA metabolism revealed complex pathways for the generation and maintenance of functional RNA in amyotrophic lateral sclerosis (ALS). Interestingly, perturbations in RNA processing have been described in ALS at various levels such as gene transcription, mRNA stabilization, transport, and translational regulations. In this paper, we will discuss the alteration of RNA profile in ALS disease, starting from transcription, the first step leading to gene expression, through the posttranscriptional regulation, including RNA/DNA binding proteins and aberrant exon splicing to protein noncoding RNAs, as lncRNA and microRNA.

## 1. Introduction

In last decade, many studies have focused on understanding the RNA metabolism and its implication in both translational and regulatory aspect of disease processes. Currently, it is proved that RNA plays a central role in many cellular processes from translating genetic information into proteins, to catalytic and regulatory aspects. Recent advances in the study of RNA metabolism revealed complex pathways in the generation and maintenance of functional RNA and in cells survival related to defects in RNA.

Mainly we may distinguish two principal families of RNA, coding (mRNA) and noncoding (miRNA and lncRNA), that are implicated in RNA metabolism that can produce cellular defects and can be causes of diseases.

Changes in gene expression and splicing patterns are described in an increasing number of complex diseases such as the neurodegeneratives [1]. Perturbations in RNA processing have been described also in ALS at various levels such as gene transcription, mRNA stabilization, and transport and translational regulation [2].

As for coding RNA, recently changes in gene expression in ALS patients have been demonstrated [3] and the discoveries of mutations in key RNA binding proteins involved in ALS have firmly placed the RNA metabolism as a central process to disease etiology [4, 5]. Moreover, pre-mRNAs alternative splicing represents an important step of posttranscriptional gene regulation by increasing the coding capacity of transcripts, and abnormalities in the RNA splicing in ALS are already known [6, 7].

About noncoding RNA, recent genome-wide analysis of the human transcriptome has revealed a plethora of long non-protein-coding RNAs (lncRNAs) whose biogenesis, regulation, and cellular roles are only starting to be elucidated. Increasing evidence points to deregulation of lncRNAs as an important, yet unexplored layer of complexity in human diseases, including neurodegenerative disorders [8]. Moreover, the literature has showed the importance of microRNAs (miRNAs), short, highly conserved noncoding RNA, that, interacting with specific sequences, can regulate mRNAs expression [9].

In this paper, we focused on RNA metabolism, starting from transcription to posttranscriptional regulation by DNA/RNA-binding proteins, noncoding RNAs as lncRNA and microRNA, and their contributions in the ALS pathogenesis.

## 2. Transcription

Alterations in gene expression have been documented in many papers in different tissues from ALS patients using microarray analysis but the data are discordant.

Two main works have been published between 2010 and 2011 that reported mRNA expression data in Peripheral Blood Mononuclear Cells (PBMCs) of ALS sporadic patients [3, 10].

Zhang and collaborators [10] performed microarray analysis on RNA extracted from PBMC of sporadic patients, and they demonstrated both activation of monocytes/macrophages and cytokines profile by the TLR4 pathways. In 2011, Mougeot and coworkers [3] carried out microarray analysis of PBMC from ALS patients and they found, as Zhang et al. [10], increased cytokines mRNAs as TNF-alpha and IL7R. Moreover, they found upregulated two genes mainly involved in ALS disease, TARDBP and SOD1, the second one already demonstrated upregulation in 2010 [11].

Concerning TDP-43 mRNA expression, Mougeot and collaborators [3] demonstrated by microarray analysis that, in PBMC from patients affected by ALS disease, TARDBP mRNA gene was upregulated by 1.5–1.8 fold-change. Additionally, Swarup and collaborators [12] discovered that TDP-43 messenger RNA was abnormally upregulated in the spinal cord of ALS subjects and its upregulation caused neuronal death by increasing of microglia neurotoxicity. In the same paper, they demonstrated that also Nuclear Factor *κ*B p65 was higher in ALS spinal cord and inhibition of NF-*κ*B in neurons overexpressing TDP-43 reduces vulnerability to toxic injury [12] suggesting a new pathway of deregulation of TDP-43 in ALS through abnormal activation of NF-*κ*B.

Interestingly, Zhang and collaborators defined by microarray experiments the main pathways related to the RNA metabolism and transcription complexes pathways, as splicing, mRNA transport, and transcription factors [10]. Also in 2010, Kudo and collaborators performed a microarray experiments on ALS mouse, cells, and human tissues [13]. As for animal model experiments, they identified in SOD1G93A blood transcriptome significant changes in the expression of the 13 genes, also regulated in SOD1G93A motor neurons or glia, supporting their use of SOD1 as a feasible clinical biomarker [13].

About mutated SOD1 cellular model, Kirby [14] showed by gene expression profiling that 268 transcripts were differently expressed in NSC34 hybridoma cells with human G93A-SOD1 mutation. They have found that transcript level of 197 genes was decreased demonstrating the important role of SOD1 mutation in transcriptional repression [14]. In particular, the genes found altered were implicated in protein degradation, immune response, and cells survival. Starting from transcription data, a first linkage between RNA expression and ALS, scientific community has been conduced to the next steps, the posttranscriptional studies, recent focus in ALS research.

## 3. Posttranscriptional Regulation

Different aspects define post-transcriptional RNA regulation; here we proposed to review some of the different steps as the role of RNA binding proteins and the splicing in altered RNA metabolism.

### 3.1. DNA/RNA Binding Proteins

The discovery of mutations in different DNA/RNA-binding proteins, as causes of both familial (FALS) and sporadic ALS (SALS), has opened enormous perspectives on the implication of post-transcriptional mechanism alterations in ALS pathogenesis.

#### 3.1.1. TAR DNA-Binding Protein 43 (TARDBP Gene)

It is homologous to the heterogeneous nuclear ribonucleoproteins (hnRNPs) [15], which are involved in RNA processing, and its abnormal cellular distribution is one of the key feature of ALS and frontotemporal lobar degeneration (FTLD) [16]. The protein is highly conserved, widely expressed, and predominantly localized to the nucleus with a very small amount being present in the cytoplasm [16, 17].

Mutations in TARDBP gene have been found in about 3 to 4% of FALS cases and in about 2% of SALS patients [4, 18]. Most TARDBP mutations are missense changes in exon 6, encoding for Gly-rich C-terminal region that allows to bind single-stranded DNA, RNA, and proteins [19–21].

The finding that TDP-43 protein aberrantly cleaved, hyperphosphorylated, and ubiquitinated is present in the cytoplasm of SALS spinal motor neurons but not in controls or in patients with Alzheimer's disease which defines the involvement of this protein in ALS [22, 23]. Moreover, both wild-type and mutant TDP-43 appear to be pathogenic, even if the role remains unclear. Different hypothesess have been formulated about the sequestering of TDP-43 in the cytoplasm, as mRNA splicing abnormalities [21] or deficit in mRNA translocation or colocalization [24–26].

Strong and collaborators have shown that TDP43 stabilizes the low molecular weight neurofilament (hNFL) mRNA through a direct interaction with the 3′-untraslated region (3′UTR) [25]. In another study Volkening et al. [26] demonstrated that the interaction of TDP-43 with the NFL mRNA 3′UTR involves ribonucleotide (UG) motifs present on stem loops of the 3′UTR.

About TDP-43 effects on other genes expression, it has been reported that TDP-43 interacts with subunits of the Mediator complex [27], which binds to the C-terminal tail of RNA polymerase II (pol II) and stimulates initiation as well as reinitiation of transcription. Also important for the regulation of gene expression is the termination of pol II: if termination is not stopped, pol II remains bound to DNA and is not available for another round of transcription. Thus, TDP-43 may exert positive as well as negative effects on transcription depending on the context.

#### 3.1.2. Ataxin-2 (ATXN2 Gene)

 It is a protein that in humans is encoded by the ATXN2 gene, which has a physiological role on mRNAs, possibly through direct RNA binding, translation, transport, and stability [28]. In 2010, Elden and collaborators [29] discovered that intermediate-length polyQ expansions (27–33 Qs) in ATXN2 significantly associated with ALS (5.5%) [29]. This finding may be very relevant in relation to its interaction with TDP-43, because both proteins are involved in RNA metabolism. In fact, Elden's data indicate that TDP-43 and Ataxin-2 can, though perhaps transiently, interact in a complex in the cytoplasm, the site of toxic function of TDP-43 in ALS, and that this interaction may depend on RNA binding. Also, ATXN2 repeats could increase its stability or inhibit its degradation, altering its concentration that promote TDP-43 pathology beyond the interactions of Ataxin-2 harboring normal repeat lengths.

#### 3.1.3. Fused in Sarcoma and Translocated in LipoSarcoma (FUS/TLS Gene)

Fused in sarcoma, and translocated in lipoSarcoma (FUS/TLS) is a heterogeneous ribonucleoprotein (hnRNP) that, as TDP-43, is involved in RNA splicing, transportation, and stabilization [30, 31]. Under physiological conditions, FUS is predominantly localized inside the nucleus; however, nuclear localization and export sequences (NLS and NES, resp.) allow the shuttling between the nucleus and the cytoplasm [32].

The C terminus that contains RNA recognition motif (RRM) allows the binding to nucleic acids, Arg-Gly-Gly (RGG) repeat rich, and zinc finger domains involved in RNA processing [32, 33].

Mutations in FUS/TLS gene have been found in 4% of FALS [4] and 0.7% of SALS patients [34].

Thus, mutations in the latter region lead to redistribution of FUS to the cytoplasm [32, 33, 35].

Subcellular localization of FUS/TLS has been studied in cells transfected with wild-type and mutant FUS/TLS gene by compartmental fractionation showing a significant increase of FUS mutants in the cytoplasmic fraction [34].

In relation to Swarup and collaborators paper [12], FUS is demonstrated to be a coactivator of NF-*κ*B, higher in ALS patients than in controls, by direct binding to NF-*κ*B subunit p65 [36]. As we mentioned in SOD1 review [32], NF-*κ*B is also a fundamental transcriptional factor involved in SOD1 expression, which already demonstrated increased in ALS patients [13].

In contrast to TDP-43, no associations of post-translational modifications of FUS such as phosphorylation, ubiquitination, or truncation have been detected in ALS or FTLD cases [37].

#### 3.1.4. Cu/Zn Superoxide Dismutase (SOD1 Gene)

 SOD1 is a soluble protein acting as a 32 kDa homodimeric enzyme to convert naturally occurring, but harmful, superoxide radicals to molecular oxygen and hydrogen peroxide. Mutations in SOD1 gene are responsible for approximately 20% of familial ALS cases [38]. With relevance to RNA metabolism, it has been demonstrated that some ALS-associated mutations can posttranscriptionally influence the expression of specific RNAs messenger [39–41]. In fact, mutSOD1s acquire new toxic functions by exerting direct RNA binding activities and consequently altering the turnover of the target mRNAs. It has been shown that the presence of mutSOD1s impairs a network of RNA-binding proteins (RBPs) and causes the destabilization of mRNA species, such as human hNFL [39] and vascular endothelial growth factor (VEGF) mRNAs [40, 41]. In particular, it has been proved that the destabilizing effects on VEGF mRNAs is mediated by direct SOD1 binding to specific adenylate/uridylate-rich elements (AREs) located in the 3′-UTR of transcripts. These cis-acting elements are specifically recognized by several transacting factors, including the embryonic lethal abnormal visual (ELAV) family of RNA-binding proteins, consisting of three neuron-specific members (HuB, HuC, and HuD) and one (HuR or HuA) that is ubiquitously expressed. These proteins bind to the ARE elements and positively regulate RNA stability and/or modulate translation of target mRNAs under certain stressful conditions, such as cytokine exposure, oxidative stress, and hypoxia. It has been demonstrated that mutSOD1 competes with HuR and HuC proteins for the same cis-acting elements, thus impairing the post-transcriptional processing of VEGF mRNA and, potentially, of other ELAVs targets. Although SOD1 protein does not contain canonical RNA-binding motifs, the gain of aberrant protein-RNA interactions can be caused by mutation-induced conformational changes. Indeed, SOD1 misfolding can lead to the exposure of normally buried polypeptide portions potentially able to bind nucleic acids. Truncation analysis of mutSOD1s showed that the RNA-binding activity resides in the N-terminal portion of the enzyme [40].

#### 3.1.5. Senataxin (SETX Gene)

 It encodes a protein that has RNA helicase activity determined by a domain at the C-terminal end of the protein. In 2004, mutations in SETX gene have been identified in ALS patients [42] but so far the role of SETX mutations in the disease remains unknown.

RNA helicases are also involved in the modification of chromatin structure, essential to the initiation of transcription, either directly through modulation of the chromatin structure, or through interactions with the assembly of the transcription-initiation complex. RNA helicases are associated with the spliceosome where they are postulated to be essential in regulating the base pairing between snRNAs and pre-mRNA [2]. The protein encoded by SETX gene contains a DNA/RNA helicase domain that regulates chromatin remodeling, thereby modulating access to the DNA template [43]. Recently, SETX was found to interact with proteins involved in transcription and pre-mRNA processing and SETX diminution resulted in defects in pre-mRNA splicing [44].

#### 3.1.6. Angiogenin (ANG)

It is a 14 kDa angiogenic ribonuclease whose activity is related to its ability in regulating ribosomal RNA (rRNA) transcription.

Human angiogenin has been shown to play a role in regulating gene expression by direct binding to DNA [45]. ANG binds to actin on the surface of endothelial cells, once bound, angiogenin is endocytosed and translocated to the nucleus of growth-stimulated endothelial cells where it accumulates in the nucleolus, binds to the promoter region of ribosomal DNA (rDNA), and stimulates rRNA transcription [46, 47]. ANG gene has been found mutated in 2.3% of FALS and 1% of SALS patients [48, 49].

Mutations in ANG gene provoke reduction of cell proliferation, angiogenic activities, and nuclear localization in ALS patients [50]; moreover, mutant ANG is toxic in motor neurons where highly expressed [51, 52].

#### 3.1.7. Chromosome 9 Open Reading Frame 72 (C9orf72)

It is a gene located on chromosome 9, composed of 10 exons and coding for 481 amino acids protein. In September 2011, two publications by Renton et al. [53] and DeJesus-Hernandez et al. [54] reported an expansion of a noncoding GGGGCC hexanucleotide repeat in intron 1 of the gene C9ORF72 associated with FTD/ALS diseases. These two papers demonstrated that the C9ORF72 repeat expansion is a very common genetic abnormality in both familial FTD (11.7%) and familial ALS (23.5%) [54]. The C9ORF72 repeat expansion was also found in 4.1% of SALS [54]. The pathogenic mechanism of the recently discovered hexanucleotide expansions in the C9ORF72 gene is not understood, it has been hypothesized that this protein is usually localized in the nuclear fraction of the cells while the presence of expansion induces this protein to shuttle in the cytoplasm compartment. As regards C9ORF72-dependent pathogenic mechanisms and RNA metabolism, 50% reduction of C9ORF72 transcript has been observed in patients with expansions [54, 55], and it has been hypothesized that expanded RNA forms pathogenic foci that trap one or more RNA binding protein(s). This mechanism of RNA toxicity, resulting in the depletion and loss of function of specific RNA binding protein(s) with affinity for the expanded RNAs, has been established in other neurological diseases [56].

### 3.2. Aberrant Exon Splicing

RNA-based mechanisms exert a vital role in governing neuronal function; consequently, alterations in RNA processing have been involved in numerous neurodegenerative conditions leading to the identification of novel pathogenic pathways. Among these, alternative splicing of pre-mRNAs represents an important step of post-transcriptional gene regulation by increasing the coding capacity of transcripts and generating proteomic diversification. Disruption of alternative splicing regulation, due to either mutations in splicing regulatory elements or aberrant activity of RNA-binding proteins, can affect the finely tuned program of gene expression and cause the abnormal production of protein isoforms. Numerous papers described abnormalities in the RNA splicing machinery in ALS. Aerbajinai and colleagues [6] demonstrated that an aberrant alternative splicing in survival motor neuron interacting protein 1 (SIP1) occurs in tissues from both ALS and spinal muscular atrophy (SMA) patients. Furthermore, a neurotoxic splice variant of peripherin (Per 61) has been described in SOD1G37R transgenic mice and in spinal cord motor neurons from two FALS cases [7]. Recently, the employment of whole-genome approaches, such as splicing sensitive microarray-based profiling, allowed to globally identify the genes and exons differentially expressed in ALS [44, 57, 58]. These analyses revealed a widespread alteration in pre-mRNA splicing in both ALS animal and cellular models and in tissues from FALS and SALS patients. The molecular bases of such alterations and their relevance to disease pathogenesis are still unclear; nevertheless, the fact that both TDP43 and FUS/TLS can act as splicing factor points to a potential involvement of these proteins in the deregulation of pre-mRNA processing observed in ALS.

## 4. Non-Protein-Coding RNA

Only 2% of the human genes encode for proteins, while the rest of the transcribed gene produces a great number of non-protein-coding RNAs (ncRNAs) as tRNA, rRNA, miRNA, natural antisense transcripts, and long noncoding RNAs (lncRNAs) [59]. Actually we are starting to study and understand the basic processes in which ncRNAs take part. Here we focus on two classes of ncRNAs, miRNA and lncRNAs.

### 4.1. MicroRNA

MiRNAs are small RNAs of 21 nucleotides and act as post-transcriptional repressors. They are able to interact with specific sequences of target mRNA (mainly in the 3′UTRs) recruiting the RNA-induced silencing complex (RISC) and, on the basis of complete or incomplete miRNA-mRNA complementary, they direct mRNA to degradation or to translational repression, respectively [60]. Two RNase III proteins, the nuclear Drosha and the cytoplasmatic Dicer, are key enzymes of the microprocessor complex: the former cleaves the pri-miRNAs into pre-miRNAs, and the latter is involved in the maturation of the pre-miRNAs [61].

It has been demonstrated that the proteins TDP-43 and FUS/TLS are able to interact physically with Drosha, suggesting their involvement in miRNA biogenesis [62]. Recently, Haramati have observed that TDP-43 facilitates the production of pre-miRNAs interacting directly with Drosha and promotes the synthesis of an miRNA subset, indispensable for neuronal outgrowth [62].

In ALS pathogenesis, mislocalization of TDP-43 due to accumulation in cytoplasmatic aggregates probably reduces the processing of TDP-43-regulated by Drosha and Dicer and generates different miRNA expression profiling.

As regards neuron survival, crucial in ALS, it has been demonstrated that ablation of Dicer in mice affects miRNA synthesis during development and leads to the loss of the ability to make functional miRNA postnatally [22]. These mutant mice show a phenotype simulating SMA with failure of functional locomotor tests, as sclerosis of the spinal ventral horns and decline in the number of motoneurons.

About ALS, miRNA profiling study on the skeletal muscle of SOD1-G93A mouse model has demonstrated that the muscle-specific miRNA miR-206 is upregulated in lower limbs of SOD1-G93A mice among 320 miRNAs tested [63].

Furthermore, in mouse model with the deletion of miR-206 it has been observed that the lack of this miRNA accelerates the disease progression, suggesting that the higher amount of miR-206, in SOD1-G93A mice may be a compensatory effect to reduce motor neurons degeneration in ALS.

All these observations sustain the importance of miRNA microprocessor complex in ALS disease, future studies on ALS patients may elucidate miRNA roles in ALS human mechanism.

### 4.2. Long Noncoding RNA (lncRNAs)

Recent genome-wide analyses of the human transcriptome have revealed a plethora of lncRNAs whose biogenesis, regulation, and cellular roles are only starting to be elucidated. LncRNAs are 300 to thousands nucleotides long that are involved in many biological processes as transcription, translation, and splicing [64].

According to the competing endogenous RNA (ceRNA) theory defined by Salmena et al., all types of RNA transcripts, coding and noncoding, could communicate with each others in a keen way supervising different cellular mechanisms [65]. For instance, it has been discovered that lncRNAs may be associated with miRNAs [65, 66].

In particular, lncRNA could act as competitor molecules able to sequester miRNA and thereby protecting target mRNA from traslational repression [67]. An example of lncRNA that may take part in splicing regulation is MALAT-1 (metastatis-associated in lung adenocarcinoma transcript), which has been found associated to nuclear-splicing phoshoproteins. Furthermore, MALAT1 is very abundant in neurons and seems to be involved in the synaptogenesis.

Increasing evidence points to deregulation of lncRNAs [68], which hopefully will show the way forward to deepen their roles in ALS disease.

## 5. Conclusions

Considering the literature data about ALS, it appears evident that this pathology has been often studied in the eyes of mitochondial or oxidative protein defects disease.

Recently, modifications of RNA metabolism have been described in ALS at various levels: splicing, editing, transport, stabilization, translation, and degradation.

In this paper, we tried to summarize the main data about the different steps of RNA metabolism, from transcription to post-transcriptional regulation and the rilevance of protein-noncoding RNA. Different genes have been found altered in gene expression profiling and often the same genes are involved in post-transcriptional pathways. Moreover, the RNA cyclelife can be controlled by protein noncoding RNA, that are playing more and more important roles in RNAs regulation.

In summary, we have tried to underline the importance of going deep to the heart of RNA metabolism since we think it may provide new insights into the complex regulation of genes expression, possibly disclosing new mechanisms involved in ALS aetiology and identifying innovative therapeutic strategies for this disease.
